# The Diagnosis of Perforated Gastric Ulcer

**Published:** 1908-10-24

**Authors:** 


### SPECIAL ARTICLE.

### THE DIAGNOSIS OF PERFORATED GASTRIC ULCER.

There used to be a time when a delay of several hours in operating upon a case of acute general peritonitis was actually recommended, the usual ground given being that the patient must be stimulated and brought round from the shock from which he was suffering. This is entirely altered now, and all statistics show that if there is one point of greater importance than another in the treatment of acute general peritonitis it is that there should be the least possible delay in having the abdomen opened. This seems to apply to all forms of acute general peritonitis. It becomes, therefore, of the very greatest importance to arrive at a diagnosis at the earliest possible moment. Every quarter of an hour's delay in getting the abdomen opened means a diminished chance of the patient's recovery.

In the first place, it is most important to look at the patient's gums, and to test the knee-jerks, at the very beginning of the examination, for lead colic, to which a blue line upon the gums would point, and a crisis of locomotor ataxy, detected by the absence of the knee-jerk. It is, of course, possible for a patient to have both plumbism or locomotor ataxy and a perforated gastric or duodenal ulcer, so that the discovery of a blue line upon the gums or of the absence of knee-jerks, must not prevent a full examination of all the other points; but every now and then either plumbism or locomotor ataxy come under observation in a guise not very unlike that of perforated gastric ulcer.

In the second place, it very seldom happens that *all* the possible symptoms and signs of perforated gastric ulcer are to be found in one individual case, and the earlier the diagnosis is arrived at, the less like the book will the symptoms and signs be. The difficulty is that there is no sign or symptom that can be regarded as pathognomonic. In one case the determining sign may be rigidity of the abdominal wall; in the next the abdomen may be perfectly supple, the nature of the lesion being diagnosed from the history and from the discovery of an impaired note in the flanks. No two cases are precisely alike, but it is most important to realise that the presence of a symptom is important; not so its absence. The diagnosis in each case must be made by weighing every symptom and sign. One is bound to be wrong sometimes, but it is important to bear in mind that laparotomy for mere stomach-ache is far less likely to cause death than is postponement of laparotomy when there is acute general peritonitis.

In the third place, it is most important not to let the abdomen absorb one's entire attention. The lungs should be thoroughly examined lest the case should be one of lobar pneumonia simulating an abdominal lesion. In some cases the trouble may seem to be abdominal when it is really within the cranium—acute meningitis with retracted abdomen and much vomiting, for example, or a cerebral tumour which has almost suddenly led to vomiting.

In the fourth place, previous to operation, it is sometimes quite impossible to distinguish between a perforated gastric ulcer and certain other lesions, such as perforated duodenal ulcer of fulminating appendicitis. The history is of much greater value than are the physical signs in making the distinction. Acute hæmorrhagic pancreatitis is another disease which may be diagnosed as perforated gastric ulcer, the true nature of the mischief only being recognised when fat necrosis is observed in the omentum after the abdomen has been opened. There is one group of conditions, however, which should not be confused with perforated gastric ulcer if their possibility is borne in mind. These are various pelvic conditions in women, such as ruptured ectopic gestation, twisted ovarian cyst-pedicle, or ruptured pyosalpinx.

After these preliminary remarks, one may say that the diagnosis of perforated gastric ulcer is arrived at by analysing carefully (1) the previous history of the patient, (2) the precise history of the present attack, (3) the general condition of the patient, and (4) the abdominal physical signs.

It is generally taught that a perforating gastric ulcer is an acute one, which has caused no symptoms previous to perforation. As a matter of fact, although a gastric ulcer may perforate in a person who has never had a gastric symptom until that moment, it is very much more common to find a history of indigestion extending back for months or years; and of these symptoms the two most constant are pain after food and vomiting. The pain may have been at any time from five minutes to two hours after the beginning of a meal, but more than half the cases have had it; and likewise, more than half the cases have been subject to attacks of vomiting. It does not follow that there has actually been a chronic ulcer causing these symptoms, but it is more common to find that a slowly spreading ulcer has ultimately extended more rapidly than the surrounding fibrosis, so that perforation and peritonitis result from it, than that the mischief is due to an acute and absolutely recent ulcer. Hæmatemesis and melæna are the exception rather than the rule in the previous history of all gastric ulcer cases; but when either has occurred, it assists considerably in the diagnosis of the acute abdominal condition. Flatulence, distension, and constipation are so common amongst patients in general that a history of them does not help much, unless it is clear that they have only developed recently and apparently without cause. If there have been attacks of epigastric pain, waking the patient at night, the argument in favour of gastric ulcer is strengthened, provided gall-stones, plumbism, and locomotor ataxy can be excluded.

The sex of the patient is of little or no assistance. It is sometimes held that a male is more likely to have a duodenal ulcer, a female a gastric ulcer; but even this is doubtful. In large collections of cases the incidence of perforated gastric ulcer is about the same in males as it is in females. Occupation seems to be of little significance either, except in so far as locality is concerned, by which we mean that gastric ulcer is far commoner in some districts than it is in others—in certain parts of the mining districts of the Midlands, for example, and perhaps in Cornwall, it is very common, and this fact has been attributed to occupation, and particularly to the perennial teapot on the hob. Age is of some slight assistance, perhaps; perforated gastric ulcer is much commoner after twenty than before it, though the women are slightly younger than the men upon the average. From 20 to 35 is the commonest age period, after which the number of cases rapidly diminishes again.

One of the most striking things about the onset of symptoms of perforated gastric ulcer is its extreme suddenness in the great majority of cases. There may or may not have been premonitory symptoms in addition to such as have already been described as previous symptoms; premonitory symptoms, however, are very variable, ranging from vague sensations of epigastric discomfort to extreme pain and hæmatemesis. Whether these have occurred or not, the patients are nearly always able to state the precise moment when the acute lesion occurred. In a few cases the perforation occurs when the patient is asleep, but in the great majority the onset is in the daytime when at work. It would seem that active digestion is one of the factors in the occurrence of the perforation; at any rate, it occurs less often in those who are fasting or in those who have eaten sparingly, than in those who have had a fairly good meal not long since; occasionally some sudden muscular effort has immediately preceded the perforation, but more often than not the patient has been doing nothing very violent at the time.

Sudden agonising pain is the invariable symptom, the localisation of which, however, varies not only in different patients, but also in the same patient at different times. Sometimes it is referred wholly or chiefly to the epigastrium, and this is perhaps its most common initial situation. At the very onset the patient may be confused as to the position of the pain, referring it now to one part of the abdomen, now to another; but it is almost invariably abdominal all the while, and most commonly epigastric at or soon after the beginning. As the hours pass and peritonitis becomes general, the patient refers the pain to the whole abdomen, with maximum intensity at the umbilicus.

Vomiting is not quite so constant an initial symptom, but it occurs in the majority of cases. Hæmatemesis is rare. The vomiting may be divided into two stages—a single bout of it at the onset of the perforation, followed by a quiescent interval, and then a recurrence of retching and vomiting when the peritonitis has become acute and general. Absence of vomiting, however, by no means excludes the diagnosis of perforated gastric ulcer, and far less stress can be laid upon it than should be upon acute agonising abdominal pain.

The general condition of the patient is extremely variable. It is but rarely that a medical man will see the case within half an hour of the onset; but it is important to realise that there are, broadly speaking, three distinct stages to be differentiated, both as regards the general condition and as regards the state of the abdomen. These three stages may be described as: (1) the early stage of obviously extreme illness; (2) the reactionary stage, during which the patient may seem to be comparatively well; and (3) the "peritonitic" stage of increasingly severe symptoms, ending in death in a limited number of hours.

When a patient has reached the third stage it is seldom difficult to decide that there is something very gravely wrong; but the aim of every medical man must be to detect the nature of the lesion before the third stage is anything like far advanced. If the patient has been seen in the first stage, and watched during the second, the very earliest signs of the third stage will convey a great deal more to the doctor who has been with the patient all the time than they would to a second doctor seeing the case for the first time. It is the second, or rallying, stage which has to be borne in mind, for it is owing to the temporary improvement that so often occurs that valuable time, and hence life, is frequently lost. Patients who have been seen in the first stage stand a much better chance of early diagnosis, operation, and cure, than do those who are first seen in the second stage; for it is only after an extensive experience of acute abdominal cases that one realises to the full the great deceptiveness of the symptoms at this time. This deceptiveness is greatly increased if any opium or morphia has been given, but nowadays it is hardly necessary to insist upon the difficulty thus caused when the diagnosis and the exact course of treatment to be followed have not already been fully decided upon.

It is clear that the general condition of the patient may be so variable that, whereas, on the one hand, it may be difficult to believe that there is little more the matter than an acute stomach ache; on the other, there may be all the signs of acute and severe illness, with anxious-drawn features, bright, glistening, but sunken, eyes; cold perspiration, clammy hands and feet, dry furred brown tongue, parched lips, a pulse that can scarcely be counted, and a general appearance of impending death, although intelligence remains perfectly clear to the end. One cannot exclude perforated gastric ulcer simply because the tongue is moist and clean, the pulse rate only 80, the temperature normal, and the features natural. Such a patient may present a very different condition a few hours later, and the points to be upon the watch for are—a desire on the patient's part to lie very still upon the back, with the knees drawn up, and with as little pressure upon the abdomen as may be; a tendency to hold the arms on the pillow above the head, particularly with the hands grasping the bed-rail; increasing dryness of the lips and tongue, of which palpation with the finger is a much better measure than is the eye; furring of the tongue without dryness is of less importance than is dryness without furring, but as time goes on the dryness is accompanied by furring, first white, and later brown; a peculiar sweetness in the smell of the patient's breath—a smell that is familiar but not easily described; a rise in the pulse rate half-hour by half-hour; a tendency to retch or vomit, or still worse, to hiccough and the occurrence of the abdominal physical signs to be described below.

The rising pulse rate is a point of extreme importance. Temperature and respiration rate matter little; the former may be normal, or slightly raised, or much depressed, without assisting the diagnosis in the least; the latter is usually unchanged, but in a few cases it is much increased—a sign of grave import, but one which is only helpful in a negative kind of way. The pulse rate, on the other hand, is so important that in no case of doubt should there be omission to have it counted and recorded hourly or half-hourly. The actual rate is not so important as is the progressive change in rate.

The abdominal physical signs are just as variable as is the general condition of the patient, and for the same reasons. It is possible to have a case in which the abdomen is neither distended nor retracted, moves well with respiration, is not tender, permitting of bimanual palpation without more than slight discomfort, and inside which it might at first seem impossible that a gastric ulcer had perforated; and yet a few hours later that same abdomen may be distended, rigid, motionless, and acutely tender. One's aim must be to operate before the latter stage is fully reached; but it is clear that one cannot operate when every sign of peritonitis is absent; the thing one has to do, therefore, is to watch for the least change in the abdominal condition, and, knowing what to look for, detect one or other of the signs of peritonitis in its earliest phase, laying more stress upon the development of one positive sign than upon the absence of several others.

Deficient freedom of abdominal movement on respiration is probably the most important of all the physical signs. The abdomen may be distended, or retracted, or neither; but in cases of perforated gastric ulcer it very soon ceases to move with respiration as freely as it ought to, and it is possible to note a progressive diminution in mobility hour by hour. Occasionally the case is seen when the upper half of the abdomen is motionless and retracted whilst the lower half still moves with respiration and is not retracted, in which case there is a characteristic furrow across the abdomen just above the umbilicus. It is always important to distinguish apparent from real movements of the wall, for in some cases the muscles may be put voluntarily into a kind of spasmodic movement by the patient, and yet there is no real expansion and retraction with respiration.

Rigidity of the abdomen is also very important, but it has to be remembered that general peritonitis may exist behind perfectly supple parietes, especially in young subjects. The rigidity is at first a uniform sense of resistance, but it rapidly increases until the abdominal wall is as "hard as a board." If this be present, peritonitis is almost certain; the difficulty is that the converse is not true; absence of board-like rigidity does not prove that there is no acute general peritonitis.

Tenderness of the abdomen is usual, but not constant; it is quite distinct from the pain which has already been discussed. Some observers lay a good deal of stress upon the presence or absence of superficial and of deep tenderness, and of the ratio of the one to the other; but it is questionable whether enough can be learned from investigating these to warrant the time required, and, above all, the suffering and questioning that the patient has to be subjected to in the investigation. General tenderness, however, is usually extreme, and the way the patient will sometimes hold out a hand to ward off the palpating fingers of the doctor is almost characteristic.

Percussion of the abdomen is often of value in one way—it serves to detect impairment of note in the flanks, due to the fluid which is very rapidly poured out into the peritoneal cavity in some of these cases. Positive evidence of this is sometimes the clinching point in a diagnosis that would otherwise be doubtful. The question of the loss of liver dulness is discussed in every text-book, and much stress is laid upon it as a sign of perforated gastric ulcer. As a matter of fact, it is of very little value indeed unless the disappearance of the liver dulness can be observed as it occurs. The mere fact that the liver dulness is deficient or absent in a given acute abdominal case means next to nothing clinically; the liver dulness is often absent, or very deficient, in many other cases, notably in patients suffering from anæmia and constipation. In perforated gastric ulcer it was supposed that free air would escape from the stomach into the peritoneal cavity, get between the liver and the abdominal wall, and so cause resonance in the hepatic region. As a matter of fact, however, resonance in this region may occur without there being free air in the peritoneal cavity; it may be due to the hepatic flexure being greatly distended with air, even when the liver still lies between the parietes and the hyper-resonant colon. Auscultatory percussion has also been noted as being of value in some cases; when free air is present in the peritoneal cavity the *bruit d'airain* may be detected by coin-tap.

Auscultation is sometimes of great assistance in deciding whether there is peritonitis or not, by detecting a friction sound over the spleen, or over the liver, or between the coils of intestine. The difficulty is that the patient involuntarily keeps the abdomen still, and the contained viscera may not be moved upon one another sufficiently to give a rub.

